# Severe and Fatal Cycling Crash Injury in Britain: Time to Make Urban Cycling Safer

**DOI:** 10.1007/s11524-022-00617-7

**Published:** 2022-03-11

**Authors:** Amanda J. Mason-Jones, Stephen Turrell, Gerardo Zavala Gomez, Caroline Tait, Robin Lovelace

**Affiliations:** 1 Department of Health Sciences, University of York, York, YO10 5DD UK; 2 Leeds City Council, Merrion House, 110 Merrion Centre, Leeds, LS2 8BB UK; 3 University of Leeds, Leeds, LS2 9JT UK

**Keywords:** Cycling, Injury, Epidemiology, Road safety, Prevention, Urban planning

## Abstract

Pedal cycling is advocated for increasing physical activity and promoting health and wellbeing. However, whilst some countries have achieved zero cyclist deaths on their roads, this is not the case for Great Britain (GB). A retrospective cross-sectional analysis was conducted of STATS19 cyclist crash data, a dataset of all police-reported traffic crashes in GB. Information about crash location, casualty, driver and vehicles involved were included as predictors of casualty severity (fatal or severe vs. slight). Sixteen thousand one hundred seventy pedal cycle crashes were reported during 2018. Severe or fatal cyclist crash injury was associated with increasing age of the cyclist (35–39 years, OR 1.38, 95% CI 1.11 to 1.73; 55–59 years, OR 1.73, 95% CI 1.35 to 2.2; 70 years and over, OR 2.87, 95% CI 2.12 to 3.87), higher road speed limits (50 MPH OR 2.10, 95% CI 1.43 to 3.07; 70 MPH OR 4.12, 95% CI 2.12 to 8.03), the involvement of goods vehicles (OR 2.08, 95% CI 1.30 to 3.33) and the months of May and June (OR 1.34 to 1.36, 95% CI 1.06 to 1.73). Urban planning that includes physical separation of pedal cyclists from other road users, raising awareness around the risks from goods vehicles and reducing road speed should be the urgent focus of interventions to increase the benefits and safety of cycling.

## Introduction

The Global Plan for a Decade of Action for Road Safety 2011–2020 sought to tackle global road deaths and progress the 50% reduction in fatalities outlined in the United Nations Sustainable Development Goals (SDGs) [1]. At least a quarter of all global injury-related deaths are the result of road traffic crashes, with people living in low- and middle-income countries disproportionately affected. However, inequality in road deaths also occurs *within* countries, with vulnerable, less affluent road users more at risk [2]. Global development is expected to increase road deaths by 2030 [2]. In the European Union (EU), 22,660 people lost their lives on roads in 2019. Although EU countries have achieved a 24% reduction in road deaths since 2010, they are highly unlikely to achieve the 50% reduction target [3].

Since the European launch of the Global Decade of Action on Road Safety, there has been a 3% reduction in the number of drivers and motorized vehicle passengers killed in road crashes, but only a 0.4% reduction in the number of people killed whilst pedal cycling (referred to as cycling in the remainder of the paper). People are considered to be vulnerable road users when cycling [4, 5], and since 2010, there have been 19,450 people killed whilst cycling on European roads with fatal cycle crashes representing 8% of all road deaths in 2018 [3]. However, differences have been reported in associated demographic and environmental factors. In the UK, in 2019, 6% of all road fatalities were people cycling [4], with socially patterned fatalities evident [6]. In Italy, cyclist fatalities largely involved people aged 65 years and above, occurred on urban roads, in collisions with cars with males more likely than females to be involved in a cycling crash [7] and more likely to be killed as a result [8]. However, research conducted in the Netherlands and Denmark found that females accounted for the majority of cycle-related deaths [3]. Detailed case analysis of 184 cyclist fatalities in Sweden explored the chain of events leading to a fatal crash. The analysis found that most of those cycling in rural areas were hit from behind, whilst those cycling in urban areas were most often involved in a crash at an intersection. The majority of fatalities took place in daylight, and involved cars, although trucks (heavy goods vehicles (HGVs) over 3500 kg) were also common, particularly in urban areas [9]. This is consistent with similar analyses of cycle crash cases in London, UK, between 2007 and 2011 [10], where the majority of fatal and severe injuries occurred on weekdays clustered around peak travel times in dry and light conditions. The most common maneuvers leading to a fatality were the vehicle turning left across the path of the person cycling and motor vehicle drivers running into the person cycling from behind. Exploratory data analysis of cycling crashes in West Yorkshire, England, found substantial geographic and social variability in the frequency of cycling crashes, with temporal analysis suggesting roads in the area had become less safe, particular for younger people cycling, between 2005 and 2012 [11].

Public health policy makers advocate cycling for increasing physical activity and promoting health and wellbeing [12, 13]. However, we should simultaneously be working towards reducing cycling-related morbidity and mortality. This study contributes to our understanding of the problem by exploring the individual, social and environmental predictors associated with fatal or severe cyclist casualties in Britain.

## Methods

We conducted a retrospective cross-sectional analysis of data available from 2018, collected by police officers, and published by the Department for Transport (DfT) on road crashes involving pedal cycles (of all types, including electrically assisted pedal cycles) in Great Britain. The STROBE reporting guidelines were followed.

### Dataset

All injuries and/or fatal crashes involving motor vehicles on UK roads must be reported to the police [14]. An administrative reporting form, known as STATS19, containing pre-determined variables (numerical, alphanumerical and text entries) is completed by the police digitally or on paper either at the scene of the crash or within a 30-day time period [14]. The STATS19 dataset is made publicly available and contains multiple variables on the circumstances of the crash, (*n* = 26) vehicle details and drivers’ demographic characteristics (*n* = 22) and demographic details and injury severity of casualties (*n* = 14).

We undertook a secondary data analysis of the crashes involving pedal cycle casualties in the STATS19 data and the associated crash and vehicle data from 2018, obtained using the free and open source STATS19 R package.

### Dependent variable

Casualty severity (fatal or severe vs. slight) was used as the binary-dependent variable. This dichotomous variable has been used previously by researchers to explore the predictors of severe and fatal road injuries [15–17]. A fatal injury was recorded when death had occurred either at the scene or within 30 days, as a result of the injuries sustained. Injuries classified as severe included, for example, spinal, vertebral or other fracture, severe head injury, unconsciousness, limb amputation and severe penetrating wounds. Slight injuries included those such as whiplash, slight shock and small cuts and/or abrasions, strains or sprains.

### Independent variables

Seventeen variables from the crash, casualty and vehicles involved were pre-selected based on their significance and importance to cyclist casualty injury severity, reported in previous research [18–20], and enabled us to explore the individual, social and environmental predictors of cyclist casualty injury severity.

Variables included time of day grouped into 4-h time periods (12:00–4:00; 4:01–08:00; 08:01–12:00; 12:01–16:00; 16:01–20:00; 20:01–23.59), day of the week (Monday to Sunday) and month of the year (January to December). Road type (single carriageway, dual carriageway, one-way-street, slip road, roundabout, unknown) and speed limit (20 to 70mph) were used. Demographic characteristics included sex of casualty (male and female), age of casualty (in years), casualty home area type (rural, small town, urban) and index of multiple deprivation (IMD) [21] of the area in which the crash took place (quintile 1 to 5). With regard to vehicle and driver involved, the vehicle type (motorcycle, car, goods vehicle, bus, taxi/private hire, others (e.g. ridden horse, tram/light rail, agricultural vehicle) and engine capacity of the vehicle (0–1000 cc; 1001–1400 cc; 1401–1800 cc; 1801–2200 cc; > 2200 cc) were included as was the sex of driver (male and female), age of driver (in years), driver home area type (rural, small town, urban) and driver IMD (quintile 1 to 5).

### Statistical analysis

R was used for data retrieval and cleaning, and IBM SPSS Statistics Version 26 was used for descriptive and logistic regression analyses. The raw data were checked for completeness and cleaned. Missing data were reported for each variable. Demographic characteristics of the cases in the dataset were explored and reported using descriptive statistics (mean and standard deviation or number and percentage).

Univariate binary logistic regression analyses were performed with casualty severity as the dichotomous-dependent variable (fatal/severe or slight injuries) and each independent variable. Thereafter, variables that were associated with the outcome (*p* < 0.05) were selected to be used in a multivariable logistic regression model. The best multivariable model was obtained with a backward procedure as this has been utilized in similar research [22]. The odds ratio was presented with 95% confidence intervals (95% CI), and statistical significance was set as *p* < 0.05.

## Results

Of the total of 16,170 cycle crash cases, the majority of cycle casualties were male (80.3%), the majority of casualties were reported in people 39 years and younger (59.8%) with a mean age of 35.9 years (SD = 16.14) and the outcome for the younger age groups tended to be slight rather than fatal/severe. The casualties were from a range of socio-economic backgrounds, though there was a higher proportion of casualties from more deprived quintile areas. Casualties were predominantly from urban home area types (89.2%) though small-town area types (5.0%) and rural settings (5.9%) were also represented in the dataset (Table 1).

**Table 1:** Demographic characteristics of the cyclist casualty and motor vehicle driver involved in the crash

	Slight		Fatal/severe		Total	
	*N*	Percentage	*N*	Percentage	*N*	Percentage
Cyclist casualty						
Female	2608	20.3%	574	17.4%	3182	19.7%
Male	10,261	79.7%	2722	82.6%	12,983	80.3%
Missing data*	5	0.0%	0	0.0%	5	0.0%
Age† (*n* = 15,825)	35.04	15.52	39.27	16.93	35.91	16.14
Age categories						
< 16 years	1765	14.1%	341	10.4%	2106	13.3%
17–24 years	1756	14.0%	372	11.4%	2128	13.4%
25–29 years	1567	12.5%	322	9.9%	1889	11.9%
30–34 years	1509	12.0%	303	9.3%	1812	11.5%
35–39 years	1198	9.5%	336	10.3%	1534	9.7%
40–44 years	1091	8.7%	306	9.4%	1397	8.8%
45–49 years	1116	8.9%	340	10.4%	1456	9.2%
50–54 years	1021	8.1%	334	10.2%	1355	8.6%
55–59 years	689	5.5%	233	7.1%	922	5.8%
60–64 years	381	3.0%	143	4.4%	524	3.3%
65–69 years	223	1.8%	94	2.9%	317	2.0%
70 years or more	242	1.9%	143	4.4%	385	2.4%
Socio-economic position						
1 (Most deprived 20%)	2590	23.2%	620	22.0%	3210	23.0%
Quintile 2	2643	23.7%	594	21.0%	3237	23.2%
Quintile 3	2148	19.3%	527	18.7%	2675	19.1%
Quintile 4	1921	17.2%	555	19.7%	2476	17.7%
5 (least deprived)	1849	16.6%	527	18.7%	2376	17.0%
Missing data*	1723	13.4%	473	14.4%	2196	13.6%
Home area type						
Rural	607	5.2%	254	8.5%	861	5.9%
Small town	552	4.7%	175	5.9%	727	5.0%
Urban	10,524	90.1%	2554	85.6%	13,078	89.2%
Missing data*	1191	9.3%	313	9.5%	1504	9.3%
Motor vehicle driver						
Female	3282	32.4%	824	30.3%	4106	32.0%
Male	6835	67.6%	1894	69.7%	8729	68.0%
Missing*	2757	21.4%	578	17.5%	3335	20.6%
Age† (*n* = 11,922)	44.18	16.2	44.36	16.69	44.22	16.31
Socio-economic position						
1 (Most deprived 20%)	1817	21.9%	465	20.7%	2282	21.7%
Quintile 2	1855	22.4%	476	21.2%	2331	22.1%
Quintile 3	1661	20.0%	482	21.5%	2143	20.3%
Quintile 4	1533	18.5%	444	19.8%	1977	18.8%
5 (least deprived)	1424	17.2%	378	16.8%	1802	17.1%
Missing data*	4584	35.6%	1051	31.9%	5635	34.8%
Home area type						
Urban	7535	86.0%	1952	81.9%	9487	85.2%
Small town	514	5.9%	186	7.8%	700	6.3%
Rural	708	8.1%	245	10.3%	953	8.6%
Missing data*	5	0.0%	0	0.0%	5	0.0%

^*^Missing data was excluded for the percentage calculation

^†^Value presented as mean and standard deviation

The majority of motor vehicle drivers involved in the cycle crashes were male (68.0%), the mean age was 44.2 years (SD = 16.31) and drivers were from a range of socio-economic backgrounds, although the highest proportion were from more deprived quintile areas. Drivers were predominantly from urban settings (85.2%) though rural home area types (8.6%) and small towns (6.3%) were also represented.

Table 2 provides information about the crash, including the road type and road speed limit and the motor vehicle involved in the crash. The highest proportion of severe/fatal crashes involving cyclists occurred on single carriageways and in 30 miles per hour (mph) (i.e. 48.3 kph) zones. The majority involved cars and happened on weekdays, during early summer and during the day between 08:00 and 20:00 h.

**Table 2:** Crash information according to the severity of the crash

	Slight		Fatal/severe		Total	
	*N*	Percentage	*N*	Percentage	*N*	Percentage
Crash information						
Road type						
Dual carriageway	968	7.5%	280	8.5%	1248	7.7%
Roundabout	1321	10.3%	337	10.2%	1658	10.3%
Single carriageway	9768	75.9%	2533	76.9%	12,301	76.1%
Slip road	85	0.7%	15	0.5%	100	0.6%
One-way street	434	3.4%	85	2.6%	519	3.2%
Unknown	298	2.3%	46	1.4%	344	2.1%
Speed limit						
20 mph	2008	15.6%	386	11.7%	2394	14.8%
30mph	9514	73.9%	2294	69.6%	11,808	73.0%
40mph	685	5.3%	220	6.7%	905	5.6%
50mph	171	1.3%	80	2.4%	251	1.6%
60mph	466	3.6%	280	8.5%	746	4.6%
70mph	30	0.2%	36	1.1%	66	0.4%
Vehicle involved						
Motorcycle	225	1.8%	48	1.5%	273	1.7%
Car	10,418	81.1%	2536	77.2%	12,954	80.3%
Goods vehicle	1084	8.4%	410	12.5%	1494	9.3%
Bus	226	1.8%	66	2.0%	292	1.8%
Taxi/private hire	655	5.1%	138	4.2%	793	4.9%
Other	232	1.8%	86	2.6%	318	2.0%
Engine capacity categories of motor vehicle involved						
0–1000 cc	727	7.6%	173	7.1%	900	7.5%
1001–1400 cc	2301	24.2%	574	23.5%	2875	24.0%
1401–1800 cc	2969	31.2%	699	28.6%	3668	30.7%
1801–2200 cc	2492	26.2%	647	26.4%	3139	26.2%
> 2200 cc	1029	10.8%	354	14.5%	1383	11.6%
Missing data	3356	26.1%	849	25.8%	4205	26.0%
Day of crash						
Monday	2060	16.0%	443	13.4%	2503	15.5%
Tuesday	2161	16.8%	533	16.2%	2694	16.7%
Wednesday	2193	17.0%	588	17.8%	2781	17.2%
Thursday	2088	16.2%	530	16.1%	2618	16.2%
Friday	2031	15.8%	517	15.7%	2548	15.8%
Saturday	1281	10.0%	364	11.0%	1645	10.2%
Sunday	1060	8.2%	321	9.7%	1381	8.5%
Month of crash						
January	924	7.2%	224	6.8%	1148	7.1%
February	767	6.0%	215	6.5%	982	6.1%
March	821	6.4%	166	5.0%	987	6.1%
April	870	6.8%	233	7.1%	1103	6.8%
May	1291	10.0%	371	11.3%	1662	10.3%
June	1377	10.7%	421	12.8%	1798	11.1%
July	1410	11.0%	344	10.4%	1754	10.8%
August	1059	8.2%	279	8.5%	1338	8.3%
September	1178	9.2%	308	9.3%	1486	9.2%
October	1166	9.1%	303	9.2%	1469	9.1%
November	1172	9.1%	247	7.5%	1419	8.8%
December	839	6.5%	185	5.6%	1024	6.3%
Time of crash						
24:00–04:00	151	1.2%	53	1.6%	204	1.3%
04:01–08:00	1422	11.0%	407	12.3%	1829	11.3%
08:01–12:00	3251	25.3%	777	23.6%	4028	24.9%
12:01–16:00	2873	22.3%	748	22.7%	3621	22.4%
16:00–20:00	4208	32.7%	1017	30.9%	5225	32.3%
20:01–23:59	969	7.5%	294	8.9%	1263	7.8%

Table 3 shows the results of the multivariable regression analysis highlighting the range of individual, vehicle and environmental predictors of fatal and severe cycle crashes.

**Table 3:** Predictors of fatal and severe cyclist crashes

	Odds ratio	95% CI	*p*
Age of cyclist casualty			
< 16 years	Reference	-	-
17–24	1.12	0.91 to 1.39	0.293
25–29	1.10	0.88 to 1.38	0.405
30–34	1.12	0.89 to 1.4	0.338
35–39	1.38	1.11 to 1.73	0.004
40–44	1.31	1.04 to 1.65	0.020
45–49	1.51	1.22 to 1.89	< 0.001
50–54	1.53	1.23 to 1.91	< 0.001
55–59	1.73	1.35 to 2.2	< 0.001
60–64	1.74	1.3 to 2.32	< 0.001
65–69	1.98	1.42 to 2.75	< 0.001
70 +	2.87	2.12 to 3.87	< 0.001
Road speed limit			
20mph	Reference	-	-
30mph	1.01	0.86 to 1.19	0.878
40mph	1.35	1.06 to 1.72	< 0.000
50mph	2.10	1.43 to 3.07	< 0.001
60mph	2.14	1.65 to 2.77	< 0.001
70mph	4.12	2.12 to 8.03	< 0.001
Time of day			
12:00–04:00	Reference	-	-
04:01–08:00	0.69	0.42 to 1.14	0.150
08:01–12:00	0.57	0.34 to 0.93	0.024
12:01–16:00	0.60	0.37 to 0.99	0.045
16:01–20:00	0.67	0.41 to 1.10	0.112
20:01–23:59	0.97	0.58 to 1.62	0.906
Month of year			
January	Reference	-	-
February	1.24	0.94 to 1.62	0.128
March	0.88	0.65 to 1.18	0.378
April	1.17	0.89 to 1.58	0.261
May	1.36	1.06 to 1.73	0.014
June	1.34	1.06 to 1.7	0.016
July	1.09	0.85 to 1.4	0.508
August	1.25	0.97 to 1.63	0.090
September	1.15	0.89 to 1.48	0.302
October	1.16	0.9 to 1.5	0.260
November	1.06	0.81 to 1.37	0.684
December	1.06	0.8 to 1.41	0.686
Day of week			
Sunday	Reference		
Monday	0.77	0.62 to 0.96	0.021
Tuesday	0.89	0.72 to 1.10	0.298
Wednesday	1.01	0.82 to 1.25	0.916
Thursday	0.90	0.73 to 1.12	0.343
Friday	0.99	0.8 to 1.23	0.934
Saturday	1.02	0.81 to 1.28	0.876
Motor vehicle type involved in the crash			
Motorcycle	Reference	-	-
Car	1.28	0.82 to 2.00	0.283
Goods vehicle	2.08	1.3 to 3.33	0.002
Bus	1.60	0.9 to 2.83	0.110
Taxi/private hire	1.17	0.7 to 1.95	0.545
Other	1.60	0.89 to 2.86	0.116

Increasing age, from 35 years old onwards, was a predictor of a fatal or severe cycle crash, with those 70 years and older with the highest odds (OR 2.87, 95% CI 2.12–3.87, *p* < 0.001). The type of vehicle with the highest odds of being involved in fatal and severe cycle casualties was goods vehicles (OR 2.08, 95% CI 1.30–3.33, *p* = 0.002). Increasing road speed limit over 30 mph (48.3 kph) was a predictor of a fatal or severe cycle crash. Roads with a speed limit of 70 mph (112.7 kph) had the highest odds of being the site of a fatal/severe cycle crash (OR 4.12, 95% CI 2.12–8.03, *p* < 0.001). With regard to time of the year, the months of May (OR 1.36, 95% CI 1.06–1.73, *p* = 0.014) and June (OR 1.34, 95% CI 1.06–1.73, *p* = 0.016) were predictors of fatal/severe cycle crashes. Mondays (OR 0.77, 95% CI 0.62–0.96, *p* = 0.021), mornings between 8am and 12 pm (OR 0.57, 95% CI 0.34–0.93, *p* = 0.024) and afternoons 12:01 pm–16:00 pm (OR 0.60, 95% CI 0.37–0.99, *p* = 0.045) were predictors of statistically significant *reduced* odds of fatal/severe cycle crashes.

## Discussion

This study explored the association between a range of individual, social and environmental variables with cycle casualty crash injury severity in the UK in 2018. We found that increasing age of casualty from 35 years onwards, being hit by a goods vehicle, road speed limit 40 MPH and over and the months of May and June were predictors of fatal or severe cycle casualties. Time of the day (between 8am and 4 pm) and day of the week (Mondays) predicted reduced odds of a fatal or severe cycling crash.

The findings are consistent with previous research exploring the impact of age on cycle injury severity and fatalities [18, 23]. Most of the cycle crashes were reported in people younger than 34 years, and there was an unacceptably high number of fatal/severe outcomes for younger people; however, in our model, casualties aged 70 and older were almost three times more likely to have a fatal or severe cycling crash outcome than those aged 16 or younger. This may be the result of increasing physical vulnerability to injury and recovery, even when crash forces are the same [24, 25], or related to physical aptitude, coordination and balance [25].

Our finding that cycle crashes with goods vehicles is a predictor or a fatal or severe injury is consistent with reports from The Royal Society for the Prevention of Accidents in relation specifically to heavy goods vehicles (HGVs) [26]. They highlighted that in London, 20% of cycle fatalities involved HGVs despite these vehicles only accounting for 4% of vehicle kilometres on London roads. Additional research in the UK has focused on the relationship between HGVs and cycling fatalities in London [10], which highlighted how urban road infrastructure and vehicle driver and cycle user awareness of their road positioning in certain situations led to a crash. However, our data suggest that the focus should be on all goods vehicles. Data on goods vehicles and cycle crashes suggests that despite the majority of goods mileage being done in non-urban environments, crashes with cycles almost always occur in urban settings. Additionally, the majority of collisions occur typically as a result of the goods vehicle overtaking a cycle and hitting them from behind, turning left across the path of the cycle or turning left with the cyclist but leaving no room for the cycle to get out of the way [26]. For HGVs in particular, the ‘direct vision standard’ and HGV safety permit introduced on London’s roads is one example of how HGVs could be made safer [27]. However, more exploration is needed to find out how to make smaller goods vehicles safer too. Urban planners, public health advocates and road safety specialists developing interventions to prevent cycle crashes should also focus on ensuring infrastructure changes that target high-risk intersections.

Increasing road speed was also a predictor of injury severity, which again is supported by previous evidence [20]. Crash injury outcomes and mortality can be significantly reduced if speeds where cycles and motorized vehicles interact remain at 30 mph (48.3 kph) or lower, supporting measures to reduce motor vehicle speeds [24, 28]. For some time now, there have been widespread calls from advocacy groups such as ‘Sustrans’ [29], to reduce speed limits, particularly in urban areas, to 20mph (32.2 kph) in order to protect vulnerable road users. In 2019 two European capital cities, Oslo and Helsinki both of which already had enviable road safety records, announced they had achieved a year with zero pedestrian or cycling fatalities. Both cities suggest the decrease in traffic speeds was the main contributor to their success [3].

Previous research exploring seasonal or monthly variations in road injuries highlight a summer peak in cycling injury hospital admissions and a drop off in the winter in terms of all crashes [30]. We found that the months of May and June were associated with increased odds of a fatal or severe cycling crash. Our findings are supported by Næss and colleagues [31], who also reported a peak in the number of cycle injury hospital admissions in Oslo, Norway, during the month of June and a seasonal increase between May and September. This particular finding needs more in-depth study. Utilising hospital admission data in the UK, Gill and Goldacre [32] also showed that whilst there is typically a lull in cycle use during the winter months, injury severity is increased.

With regard to time of the day, we found that the hours between 8am and 4 pm predicted *reduced* odds of a severe or fatal cycling crash. This may be because of reduced vehicle speeds during peak hours and increased visibility during the daylight hours. A previous study in Spain, exploring determinants of fatal and severe cycle crashes, found that the majority of cycling fatalities took place at night or early morning during darkness [19]. This may reflect different environmental infrastructure such as the lighting available on the roads, as increasing street lighting has been shown to reduce injury severity in both urban and rural settings in Canada [18].

The major strength of this study is the inclusion of all reported cycle crash incidents in Great Britain (GB), and wide range of individual, social and environmental variables included, which can be generalized to GB. The data are collected via a consistent reporting method by trained police officers. A limitation of this study is that it represents 1 year of data, and thus the authors could not examine annual trends. Furthermore, whilst the STATS19 dataset is relatively complete for crashes that are reported to police, it is acknowledged that a large number of crashes are not reported to police, particularly when involving cycles [33, 34] and a potential source of bias could be the underestimation of incidents where injury is regarded as ‘slight’ [35, 36].

The findings from this study offer considerable insight for public health specialists, urban planners, policy makers, transport officials and the general public into the factors that predict the severity of injury outcome for cycle casualties. The study identifies key factors to consider from a prevention and emergency response perspective. This is particularly timely given the increased funding and emphasis being placed on sustainable methods of transport during the COVID-19 pandemic [37, 38]. The inclusion of data on socio-economic status of casualties and drivers is a unique and valuable contribution regarding the wider determinants of crash severity. However, in order to produce the most reliable and important information for decision makers, police forces and local authorities should seek to minimize the amount of missing data as a priority and include data from trauma registries [39].

Future research should look to combine the data from datasets similar to STATS19 with data from insurance companies and emergency departments to provide the most complete picture of road crash information so that more in-depth investigation of the significant predictors of fatal and severe cycle crash injuries can be undertaken.

## Conclusion

Reducing the incidence and severity of cycling crashes should be an absolute priority for promoting urban health. This study provides a unique contribution that explores individual, social and environmental predictors of cycle crash injury severity. The popularity of online shopping has resulted in more goods delivery vehicles on the roads. Therefore, preventing fatalities as a result of cycle and goods vehicle crashes is especially important. We suggest a comprehensive holistic approach that includes urban planning and improving infrastructure to prevent fatalities and severe injury of people who cycle including separating them physically by providing more dedicated cycle paths, reducing road speed limits where cycles and motorized vehicles are likely to interact, technical improvements to goods vehicles and targeted education and awareness raising to improve our understanding of the risks posed in certain situations and how they can be mitigated.
