# Correction to: Severe and Fatal Cycling Crash Injury in Britain: Time to Make Urban Cycling Safer

**DOI:** 10.1007/s11524-022-00636-4

**Published:** 2022-04-04

**Authors:** Amanda J. Mason-Jones, Stephen Turrell, Gerardo Zavala Gomez, Caroline Tait, Robin Lovelace

**Affiliations:** 1 Department of Health Sciences, University of York, York, YO10 5DD UK; 2 Leeds City Council, Merrion House, 110 Merrion Centre, Leeds, LS2 8BB UK; 3 University of Leeds, Leeds, LS2 9JT UK

**Correction to: J Urban Health**

**https://doi.org/10.1007/s11524-022-00617-7**

In this article as originally published, Results paragraph two last sentence labelled the drivers’ home area incorrectly. The drivers were predominantly from urban settings (85.2%) though rural home area types (8.6%) and small towns (6.3%) were also represented. In Table 1 the first label in the motor vehicle driver home area type should read urban, then small town and then rural.

The original article has been corrected.

